# Protein Kinase CK2 Regulates Nerve/Glial Antigen (NG)2-Mediated Angiogenic Activity of Human Pericytes

**DOI:** 10.3390/cells9061546

**Published:** 2020-06-25

**Authors:** Beate M. Schmitt, Anne S. Boewe, Vivien Becker, Lisa Nalbach, Yuan Gu, Claudia Götz, Michael D. Menger, Matthias W. Laschke, Emmanuel Ampofo

**Affiliations:** 1 Institute for Clinical & Experimental Surgery, Saarland University, 66421 Homburg, Germany; beate.schmitt@uks.eu (B.M.S.); Anne.boewe@uks.eu (A.S.B.); Vivien.becker@uks.eu (V.B.); lisa.nalbach@uks.eu (L.N.); Yuan.gu@uks.eu (Y.G.); michael.menger@uks.eu (M.D.M.); matthias.laschke@uks.eu (M.W.L.); 2 Medical Biochemistry and Molecular Biology, Saarland University, 66421 Homburg, Germany; claudia.goetz@uks.eu

**Keywords:** CK2, NG2, promoter, pericytes, endothelial cells, angiogenesis

## Abstract

Protein kinase CK2 is a crucial regulator of endothelial cell proliferation, migration and sprouting during angiogenesis. However, it is still unknown whether this kinase additionally affects the angiogenic activity of other vessel-associated cells. In this study, we investigated the effect of CK2 inhibition on primary human pericytes. We found that CK2 inhibition reduces the expression of nerve/glial antigen (NG)2, a crucial factor which is involved in angiogenic processes. Reporter gene assays revealed a 114 bp transcriptional active region of the human NG2 promoter, whose activity was decreased after CK2 inhibition. Functional analyses demonstrated that the pharmacological inhibition of CK2 by CX-4945 suppresses pericyte proliferation, migration, spheroid sprouting and the stabilization of endothelial tubes. Moreover, aortic rings of NG2^−/−^ mice showed a significantly reduced vascular sprouting when compared to rings of NG2^+/+^ mice, indicating that NG2 is an important regulator of the angiogenic activity of pericytes. In vivo, implanted Matrigel plugs containing CX-4945-treated pericytes exhibited a lower microvessel density when compared to controls. These findings demonstrate that CK2 regulates the angiogenic activity of pericytes through NG2 gene expression. Hence, the inhibition of CK2 represents a promising anti-angiogenic strategy, because it does not only target endothelial cells, but also vessel-associated pericytes.

## 1. Introduction

Protein kinase CK2 is an ubiquitously expressed serine/threonine kinase consisting of two catalytic CK2α and CK2α’ subunits as well as two regulatory CK2β subunits [1]. To date, more than five hundred proteins have been identified as substrates of CK2, including structure proteins, membrane proteins and transcription factors [2]. CK2 is involved in various cellular processes, including thrombus formation, insulin secretion and angiogenesis [3,4,5,6]. Hence, a broad spectrum of CK2 inhibitors was developed in recent decades [7]. Commonly used CK2 inhibitors are 2-dimethylamino-4,5,6,7-tetrabromo-1H-benzimidazole (DMAT) [8], 4,5,6,7-tetrabromobenzotriazole (TBB) [9] and CX-4945 [10]. The latter is orally bioavailable [11] and has entered phase II clinical trials (ClinicalTrials.gov Identifier: NCT02128282).

There is a growing number of studies reporting that, besides endothelial cells, pericytes have a leading function in supporting vascular sprouting and blood vessel formation [12]. Pericytes are embedded within the basal membrane of small arterioles, capillaries and venules [13]. They interact directly with neighboring endothelial cells and the extracellular matrix (ECM), leading to the activation of pro- or anti-angiogenic pathways [14,15]. In recent decades, several surface proteins on pericytes have been identified, which contribute to their angiogenic potential [13]. One of these is nerve/glial antigen (NG)2, also known as chondroitin sulphate proteoglycane 4 (CSPG4), high molecular weight melanoma-associated antigen (HMW-MAA) or melanoma chondroitin sulfate proteoglycane (MCSP) [16,17,18]. NG2 consists of a large, posttranslational modified extracellular domain, which interacts with β1-integrin (CD29), resulting in enhanced cell proliferation [19,20]. Moreover, the extracellular domain binds to collagen VI [21,22,23], and thus promotes the detachment of pericytes from the vascular wall and their migration into the surrounding tissue as a major prerequisite for angiogenic sprout formation [24]. Accordingly, the loss of NG2 reduces the angiogenic potential of pericytes [25].

In the present study, we analyzed, for the first time, the regulatory function of CK2 on NG2-mediated angiogenic activity of primary human pericytes. We found that inhibition of CK2 suppresses the ability of pericytes to proliferate, migrate and stabilize endothelial tubes. Additional analyses revealed that CK2 inhibition reduces NG2 gene expression. Vascular sprouting out of aortic rings from wild type (NG2^+/+^) and NG2 knockout (NG2^−/−^) mice further confirmed the suppressive effect of CK2 inhibition on the angiogenic activity of pericytes. These in vitro results were finally verified by means of an in vivo Matrigel plug assay. 

## 2. Materials and Methods

### 2.1. Chemical and Biological Reagents

Pericyte growth medium (PCGM) and endothelial growth medium were from PromoCell (Heidelberg, Germany). Dulbecco's Modified Eagle's Medium (DMEM), Lipofectamine3000 reagent, PureLink^TM^ Genomic DNA Mini Kit and the restriction enzymes HindIII and XhoI were from Thermo Fisher Scientific (Karlsruhe, Germany). Matrigel was from Corning (Wiesbaden, Germany). Vascular endothelial growth factor (VEGF) and basic fibroblast growth factor (bFGF) were from R&D systems (Wiesbaden, Germany). Heparin was from Braun (Melsungen, Germany). Methylcellulose, Cycloheximide (CHX) and PKH67 green fluorescence linker were from Sigma-Aldrich (Taufkirchen, Germany). CX-4945 was from ActivateScientific (purity > 95%, Prien, Germany), TBB and bovine serum albumin (BSA) from Santa Cruz Biotechnology (Heidelberg, Germany). 4′,6-Diamidine-2′-phenylindole dihydrochloride (DAPI) and Bromdesoxyuridin (BrdU) was from Roche (Mannheim, Germany). Cell lysis reagent QIAzol was purchased from Qiagen (Hilden, Germany). The qScriber™ cDNA Synthesis Kit, ORA™ SEE qPCR Green ROX L Mix and SampleIN^TM^ Direct PCR Kit were from HighQu (Kraichtal, Germany). The set of three small interfering RNA (siRNA) duplexes directed against CK2α, CK2α’ and non-targeting siRNA control (ON-TARGET plus SMARTpools) was from GE Healthcare Dharmacon (Freiburg, Germany). Luciferase Assay System was from Promega (Walldorf, Germany). The NG2-plasmid (pEF6-CSPG4-myc-his) was from addgene (Watertown, MA, USA). 

### 2.2. Antibodies

The anti-NG2 antibody (sc-166251) and CK2β antibody (E9) were from Santa Cruz Biotechnology. The anti-α-tubulin (B-5-1-2/T5168) antibody was from Sigma-Aldrich. The anti-Akt1/2/3 antibody (11E7) was from Cell Signaling (Frankfurt am Main, Germany). The anti-CK2α #26 and anti-CK2α’ #30 antibodies were generated as described previously [26]. The human anti-CD31 antibody and anti-AKT1 (phospho S129) antibody [EPR6150] was from Abcam (Cambridge, UK). The mouse anti-CD31 antibody (DIA310) was from Dianova (Hamburg, Germany). The peroxidase-labeled anti-rabbit antibody (NIF 824) and peroxidase-labeled anti-mouse antibody (NIF 825) were from GE healthcare (Freiburg, Germany). The flow cytometry antibodies anti-CD29 (555443), anti-activated CD29 (556049), anti-CD31 (555446), anti-chondroitin sulfate proteoglycan 4 (NG2) (562415) and IgG1-κ isotype control (555749) were from BD Biosciences (Heidelberg, Germany). The BrdU antibody (BU20A) was from eBioscience™ Fisher Scientific (Schwerte, Germany).

### 2.3. Cell Culture and Treatment 

Primary human placenta-derived pericytes, primary normal human dermal fibroblasts (NHDF) and human umbilical vein endothelial cells (HUVEC) from PromoCell were cultivated at 37 °C under a humidified 95%/5% (vol/vol) mixture of air and CO_2_. The cells were passaged at a split ratio of 1:3 after reaching confluence. All experiments were carried out with confluent cells between the third and seventh passage. They were treated with 10 µM CX-4945, 50 µM TBB or vehicle (DMSO) for 48 h before the analyses. 

### 2.4. Water-Soluble Tetrazolium (WST)-1 Assay

A WST-1 assay (Roche, Mannheim, Germany) was used to evaluate the mitochondrial activity as a parameter of cell viability of pericytes, as described previously in detail [27].

### 2.5. Lactate Dehydrogenase (LDH) Assay

An LDH assay (Cytotoxicity Detection KitPLUS, Roche) was used to analyze the cytotoxic effects of CX-4945 on pericytes, as previously described in detail [27]. Briefly, pericytes were treated with vehicle (DMSO) or CX-4945 (10 µM) for 48 h. For the high toxicity control (HC), pericytes were incubated with a lysis reagent containing Triton-X100 (0.1%) to permeabilize their cell wall. To investigate the LDH release, pericytes were then incubated with the Dye-Catalyst mixture and analyzed with a microplate reader. 

### 2.6. Scratch Assay

The migratory capacity of pericytes was assessed by a scratch assay. For this purpose, pericytes were seeded in a 24-well plate. After reaching confluence, the cell monolayer was scratched with a pipette tip and then rinsed twice with phosphate-buffered saline (PBS) to remove non-adherent cells. Phase-contrast light micrographs were taken immediately (0 h), as well as 16 and 24 h after scratching. The gap area was measured by means of ImageJ software; version 1.52a (U.S. National Institutes of Health (NIH), Bethesda, MD, USA).

### 2.7. Growth Curves

Pericytes (2 × 10^6^ cells per well) were seeded in a 24-well plate and cultivated overnight. Then, the cells were treated with 10 µM CX-4945 or vehicle for 24 and 48 h. Thereafter, they were detached, centrifuged and suspended with fresh culture medium. Aliquots of the suspended cells were stained with trypan blue (0.4%) and counted by a LUNA™ Automated Cell Counter according to the manufacturer's protocol. 

### 2.8. Transwell Migration Assay

The migratory activity of pericytes exposed to vehicle or 10 µM CX-4945 for 48 h was assessed using 24-well chemotaxis chambers and polyvinylpyrrolidone-coated polycarbonate filters with a pore size of 8 mm (BD Biosciences, Heidelberg, Germany). After overnight incubation (37 °C, 5% CO_2_) of the filters in PCGM without any supplements, medium was removed and 750 µL PCGM supplemented with 5% FCS was added to each of the lower wells and 500 µL PCGM/0.1% FCS containing 2.5 × 10^5^ treated cells were added to the upper well. After 5 h cultivation, non-migrated cells were removed from the upper surface of the filters by cotton swabs. Migrated cells (adherent to the lower surface) were fixed with methanol and stained with Dade Diff-Quick (Dade Diagnostika GmbH, München, Germany). The number of migrated cells was counted in 20 microscopic regions of interest at 20× magnification (BZ-8000; Keyence, Osaka, Japan).

### 2.9. Spheroid Sprouting Assay

For the generation of spheroids, pericytes were suspended in medium containing 0.24% (*w/v*) methylcellulose and seeded (500 cells/100 µL) in non-adherent, round-bottom, 96-well plates (Greiner Bio-One, Frickenhausen, Germany). After incubation for 24 h, the newly developed spheroids were collected and resuspended in collagen solution, as previously described in detail [28]. After 48 h of incubation with 10 µM CX-4945 or vehicle, the spheroid-sprouting capacity was quantified by measuring the cumulative length of sprouts.

### 2.10. Tube Formation Assay

To assess the ideal ratio of pericytes and HUVEC for tube formation in co-culture experiments, both cell types were first seeded in different ratios (1:4 and 1:10) in a 96-well plate (overall cell number: 1.5 × 10^4^ per well), which contained 50 µL Matrigel. In a second set of experiments, pericytes were pretreated with 10 µM CX-4945 or vehicle for 48 h. Thereafter, the cells were washed twice with PBS and stained with PKH67 green fluorescence linker according to the manufacturer's instructions. Then, the pretreated pericytes were co-cultivated with HUVEC in a ratio of 1:10. Phase-contrast light micrographs and fluorescence images were taken after 6 or 24 h. Tube formation was quantified by measuring the number of tube meshes (i.e., areas completely surrounded by endothelial tubes) per high power field (HPF) using ImageJ software; version 1.25a (U.S. NIH, Bethesda, MD, USA).

### 2.11. NG2 Promotor Analyses 

Genomic DNA was isolated from primary human pericytes by means of PureLinkTM Genomic DNA Mini Kit according to the manufacturer’s instructions. A region of 2677 bp in front of the NG2 gene was amplified and fragmented by polymerase chain reaction (PCR) (for primer sequences see Table S1). All expression plasmids were derived from the pGL4.10 basic vector. The NG2 promotor fragments were cloned by XhoI and HindIII digestion and verified by DNA sequencing (Eurofins Genomics, Ebersberg, Germany). 

### 2.12. Reporter Gene Assay

The transcriptional activity of the putative NG2 promoter fragments was assessed by reporter gene assays according to the manufacturer’s instructions. Briefly, HEK cells were transfected with the generated pGL4.10 expression vectors by means of Lipofectamine3000 for 24 h. After incubation, the cells were immediately lysed or incubated with 10 µM CX-4945 or vehicle (DMSO) for an additional 24 h. Luciferase activity was detected by a luminescence plate reader.

### 2.13. Cell Transfection

Pericytes were transfected with siRNA duplexes (100 nM) directed against CK2α and CK2α’ or with control (ctrl)-siRNA using HiPerFect transfection reagent for 48 h. For NG2 overexpression, the cells were transfected with pEF6-CSPG4-myc-his or a ctrl-plasmid using Lipofectamine3000 reagent for 24 h. Subsequently, the cells were used for flow cytometry or Western blot analyses.

### 2.14. Western Blot Analysis

Whole cell or tissue extracts were separated through a 7.5% or 12.5% SDS polyacrylamide gel and transferred onto a polyvinylidene difluoride (PVDF) membrane. The membrane was blocked with 5% dry milk in Tris-buffered saline TBS (0.1% Tween20) for 1 h and then incubated with the primary antibodies (anti-CK2α, anti-CK2α’, anti-CK2β, anti-NG2, anti-pAkt, anti-Akt and anti-α-tubulin; dilution 1:500) in TBS (0.1% Tween20) containing 1% dry milk overnight (4 °C). The membrane was incubated with a peroxidase-coupled secondary antibody (anti-rabbit 1:1500 or anti-mouse 1:2000) for 1 h. Protein expression was visualized by luminol-enhanced chemiluminescence (ECL; GE Healthcare).

### 2.15. Flow Cytometry

Cells were washed in PBS and harvested by scratching. Subsequently, the cells were incubated with the phycoerythrin (PE)-labeled primary antibodies and the PE-labeled IgG control antibodies for 1 h at room temperature. Then, the cells were washed in PBS and the mean fluorescence intensity (MFI) of 5000 cells was analyzed in the FL-2 channel by a FACScan flow cytometer (Becton Dickinson, San Antonio, USA) using the CellQuest software (version 5.1). 

Proliferative cells were detected by a BrdU-assay (Thermo Scientific Bremen, Germany) according to the manufacturer’s protocol. Briefly, pericytes were incubated with BrdU. Then, the cells were washed, fixed and permeabilized. BrdU was detected by using a BrdU-antibody provided by the manufacturer. The MFI of 5000 cells was analyzed in the FL-1 and FL-2 channel by a FACScan flow cytometer using the CellQuest software (version 5.1).

### 2.16. Quantitative Real-Time PCR (qRT-PCR)

RNA was isolated by using QIAzol lysis reagent and cDNA was prepared by means of qScriber™ cDNA Synthesis Kit. The amount of mRNA was analyzed by qRT-PCR using ORA™ SEE qPCR Green ROX L Mix according to the manufacturer's instructions. Forward and reverse primers were used at a concentration of 500 nM. Data collection and analyses were performed by MiniOpticon Real-Time PCR Detection System and the 2^−ΔΔCt^ method.

### 2.17. Animals

All experiments were approved by the local governmental animal protection committee (Landesamt für Verbraucherschutz, Abteilung C Lebensmittel und Veterinärwesen, Saarbrücken, Germany with the approval number: 012019) and were conducted in accordance with the European legislation on protection of animals (Directive 2010/63/EU) and the NIH Guidelines for the Care and Use of Laboratory Animals (http://oacu.od.nih.gov/regs/index.htm. 8th Edition; 2011).

Homozygous NG2-CreERT2 knock-in mice (TgH(NG2-CreERT2)) [29] were crossbred with reporter mice (B6;129S6-Gt(ROSA)26Sor^tm9(CAG-tdTomato)Hze/J^ (Ai14)) [30]. Homozygous NG2-CreERT2xRosa26-tdTomato mice lacking NG2 (NG2^−/−^) [29] as well as C57BL/6N wild type mice (NG2^+/+^) with a body weight of 25–35 g were used as donors for aortic rings. For Matrigel plug assays, 8- to 12-week-old male CD1 nu/nu mice with a body weight of 25–30 g were used. 

### 2.18. Genotyping

To distinguish between NG2^−/−^ and NG2^+/+^ mice, we used SampleIN^TM^ Direct PCR Kit. Genomic DNA was extracted from ear notches and then subjected to PCR using a designed primer set: Genotypes (NG2 wt allele: 557 bp and mutant allele: 829 bp) were determined by resolving PCR products by means of agarose gel electrophoresis.

### 2.19. Aortic Ring Assay

Aortic rings of NG2^+/+^ and NG2^−/−^ mice were embedded in Matrigel. After 1 h, the Matrigel was polymerized and DMEM supplemented with 10% FCS was added. The aortic rings were then incubated at 37 °C for 6 days with 10 µM CX-4945 or vehicle (DMSO), including a medium change on day 3. The rings were analyzed by phase-contrast microscopy to assess the sprouting area. 

### 2.20. Matrigel Plug Assay

Matrigel plugs consisting of 150 µL endothelial growth medium were mixed with the same volume of growth factor-reduced Matrigel (~20 mg/mL) containing VEGF (2 µg/mL), bFGF (2 mg/mL) and heparin (100 IU/mL) were subcutaneously injected into NG2^+/+^ and NG2^−/−^ mice. Moreover, pericytes were treated with 10 µM CX-4549 or vehicle for 48 h. Thereafter, the cells were washed twice with PBS. Treated pericytes (1.5 × 10^4^ cells) and HUVEC (1.5 × 10^5^ cells) in a total volume of 150 µL endothelial growth medium were mixed with the same volume of growth factor-reduced Matrigel (~20 mg/mL) containing VEGF (2 µg/mL), bFGF (2 mg/mL) and heparin (100 IU/mL). Then, 300 µL Matrigel admixed with the cell suspension were subcutaneously injected into CD1 nu/nu mice. After 7 days, the Matrigel plugs were isolated for immunohistochemical analyses. 

### 2.21. Immunohistochemistry

Paraformaldehyde-fixed specimens of Matrigel plugs were embedded in paraffin for the cutting of 3 µm-thick sections. The sections were stained with a human anti-CD31 antibody followed by the corresponding secondary-coupled fluorescence antibody. As negative controls, we used sections solely incubated with the secondary antibody. Microvessel density was quantified by counting human CD31-positive microvessels in six HPFs of each plug at 200× magnification (BX60, Olympus, Hamburg, Germany).

### 2.22. Statistical Analysis

All in vitro and in vivo experiments were reproduced at least three times. For in vivo studies, we used five animals per group and no mice were excluded from the statistical analysis. After testing the data for normal distribution and equal variance, differences between two groups were assessed by the unpaired Student's *t-*test. To test differences between multiple groups, one way ANOVA was applied. This was followed by the Tukey post-hoc test by means of Prism software 8 (GraphPad). All values are expressed as mean ± SD. Statistical significance was accepted for *p* < 0.05.

## 3. Results

### 3.1. Effect of CK2 Inhibition on NG2 Expression

**Figure 1:**
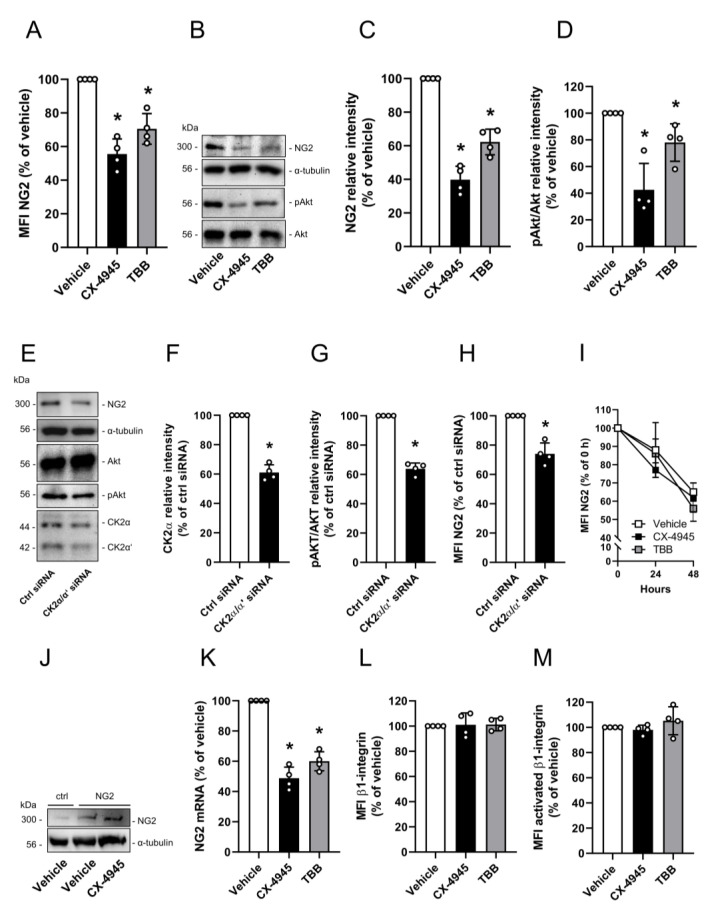
Effect of CK2 inhibition on NG2 expression. (**A**) Pericytes were treated with vehicle (DMSO), CX-4945 (10 µM) or TBB (50 µM) for 48 h. The cells were scratched and the mean fluorescence intensity (MFI) of NG2-positive cells was assessed by flow cytometry. Vehicle-treated cells were set 100%. Mean ± SD. * *p* < 0.05 versus vehicle (*n* = 4). (**B**–**D**) Pericytes were treated as described in (**A**). The cells were lysed and the expression of NG2, Akt, pAkt and α-tubulin (as loading control) was analyzed by Western blot (**B**). NG2/α-tubulin (**C**) and pAkt/Akt (**D**) were assessed by quantitative analysis of Western blots. Vehicle-treated cells were set at 100%. Mean ± SD. * *p* < 0.05 versus Vehicle (*n* = 4). (**E**–**H**) Pericytes were treated with a combination of CK2α and CK2α’ siRNA or with ctrl siRNA. The cells were lysed and the expression of NG2, Akt, pAkt, CK2α, CK2α’ and α-tubulin (as loading control) was analyzed by Western blot (**E**). CK2α/α-tubulin was assessed by quantitative analysis of Western blots (**F**). pAkt/Akt was assessed by quantitative analysis of Western blots (**G**). MFI of NG2-positive cells was detected by flow cytometry (**H**). ctrl siRNA-treated cells were set as 100%. Mean ± SD. * *p* < 0.05 versus ctrl siRNA (*n* = 4). (**I**) Pericytes were treated with vehicle (DMSO), CX-4945 (10 µM) or TBB (50 µM) in the presence of Cycloheximide (CHX) for 0, 24 and 48 h. Then, the cells were scratched and the MFI of NG2-positive cells was assessed by flow cytometry. Cells at 0 h were set 100%. Mean ± SD (*n* = 4). (**J**) Pericytes were transfected with ctrl-plasmid or NG2-plasmid for 24 h following treatment with vehicle or CX-4945. The cells were lysed and the expression of NG2 and α-tubulin (as loading control) was analyzed by Western blot. (**K**) Pericytes were treated as described in (**A**) and total RNA was isolated. The relative gene expression of NG2 was examined by qRT-PCR normalized to GAPDH as housekeeping gene. Vehicle-treated cells were set 100%. Mean ± SD. * *p* < 0.05 versus vehicle (*n* = 4). (**L** and **M**) Pericytes were treated as described in (**A**) and the MFI of β1-integrin (**L**) and activated β1-integrin (**M**) was assessed by flow cytometry. Vehicle-treated cells were set as 100%. Mean ± SD (*n* = 4).

First, we investigated the effect of CK2 inhibition on NG2 expression. The treatment of pericytes with CX-4945 or TBB significantly reduced the protein levels of NG2 when compared to controls, as shown by flow cytometry (Figure 1A) and Western blot analyses (Figure 1B,C). In addition, CX-4945 and TBB treatment diminished the CK2-dependent phosphorylation of Akt on serine 129 (pAkt) (Figure 1B,D). Moreover, silencing of the catalytic subunits CK2α and CK2α’ resulted in reduced protein levels of NG2 and pAkt (Figure 1E–H). To assess whether CK2 inhibition affects NG2 protein stability, pericytes were treated with vehicle or the two CK2 inhibitors CX-4945 and TBB in the presence of the CHX, which is an inhibitor of protein translation. NG2 protein levels progressively decreased throughout an observation period of 48 h without any significant differences between the groups indicating that CK2 inhibition does not affect NG2 protein stability (Figure 1I). This finding was confirmed by the observation that NG2 overexpression in pericytes is not altered by the inhibition of CK2 activity (Figure 1J). On the other hand, CK2 inhibition significantly reduced NG2 mRNA levels (Figure 1K). Accordingly, it can be concluded that the kinase regulates NG2 gene expression. 

β1-integrin binds to NG2 and the interaction of the two proteins mediates signal transduction, resulting in cell proliferation [31]. To exclude the fact that CK2 inhibition also affects β1-integrin, we determined the expression of this surface protein after CX-4945 and TBB treatment. We found that neither the protein levels nor the activation of β1-integrin is affected by CK2 inhibition (Figure 1L,M).

### 3.2. Effect of CK2 Inhibition on NG2 Promoter Activity 

**Figure 2:**
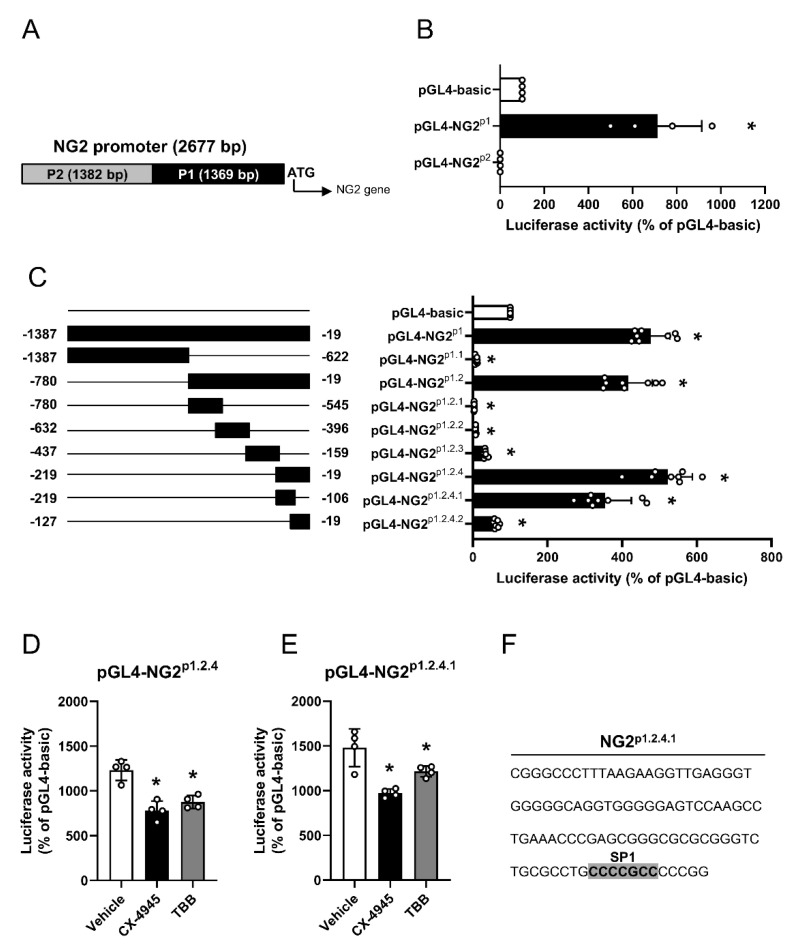
Characterization of the NG2 promotor region and identification of putative transcription factor binding sites. (**A**) Schematic illustration of the 2677 bp NG2 promoter region in front of the translational start, which contains the two overlapping truncated fragments P1 (1369 bp) and P2 (1382 bp). (**B**) HEK cells were transfected with pGL4-basic, pGL4-NG2^p1^ or pGL4-NG2^p2^ and the transcriptional activity was detected after 48 h by means of luciferase activity. Luciferase activity of pGL4-basic-transfected cells was used as control and set as 100%. Mean ± SD. * *p* < 0.05 versus pGL4-basic (*n* = 4). (**C**) HEK cells were transfected with the indicated truncated fragments of pGL4-NG2^p1^ and the transcriptional activity was detected after 48 h by means of luciferase activity. Luciferase activity of pGL4-basic-transfected cells was used as control and set 100%. Mean ± SD. * *p* < 0.05 versus pGL4-basic (*n* = 8). (**D** and **E**) HEK cells were transfected with pGL4-NG2^p1.2.4^ (**D**) or pGL4-NG2^p1.2.4.1^ (**E**) for 24 h and treated with vehicle (DMSO), CX-4945 (10 µM) or TBB (50 µM). After 24 h, transcriptional activity was determined by luciferase activity. Luciferase activity of pGL4-basic-transfected cells was used as control and set as 100%. Mean ± SD. * *p* < 0.05 versus vehicle (*n* = 4). (**F**) Nucleotide sequence of NG2^p1.2.4.1^ (114 bp). The SP1 consensus sequence is highlighted in grey.

The human NG2 promoter differs from the murine NG2 promoter in its chromosomal localization [32]. To characterize the human NG2 promotor, we first determined a 2677 bp region upstream of the NG2 start codon as a putative transcriptional active region (Figure 2A). To assess the activity of this region, we generated overlapping fragments (NG2^p1^ and NG^p2^), which were cloned into the luciferase vector pGL4. Of interest, we only detected an enhanced transcriptional activity of NG2^p1^, as shown by an increased luciferase signal (Figure 2B). Accordingly, we stepwise generated overlapping truncated fragments of NG2^p1^ (Figure 2C). Out of these fragments, NG2^p1.2.4.1^ was finally identified as the enhancing region of the NG2 promoter (Figure 2C). 

To investigate the effect of CK2 inhibition on NG2 promotor activity, HEK cells were transfected with the luciferase vectors pGL4-NG2^p1.2.4^ or pGL4-NG2^p1.2.4.1^ and subsequently treated with CX-4945, TBB or vehicle. As expected, we detected a significantly decreased luciferase activity after CK2 inhibition when compared to vehicle-treated controls (Figure 2D,E). In silico analyses of NG2^p1.2.4.1^ revealed a consensus site for the transcription factor SP1 (Figure 2F).

### 3.3. Effect of CK2 Inhibition on Proliferation, Migration and Sprouting of Pericytes

**Figure 3:**
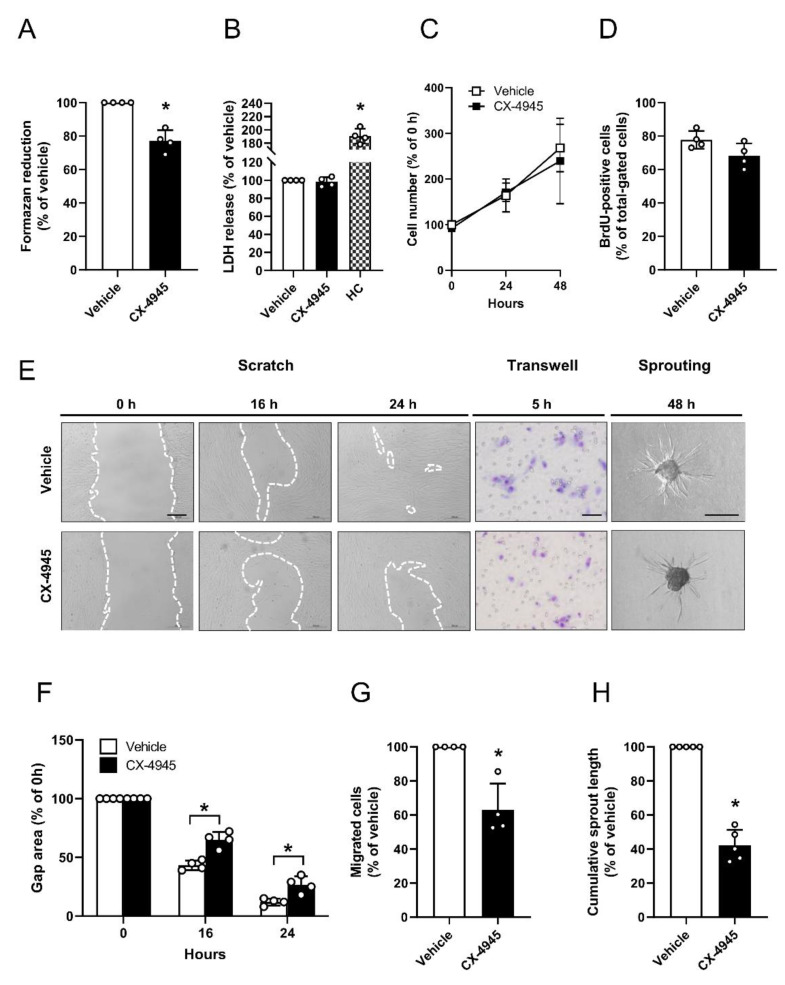
Effect of CK2 inhibition on proliferation, migration and sprouting of pericytes. (**A**) Pericytes were treated with vehicle (DMSO) or CX-4945 (10 µM) for 48 h and the mitochondrial activity was analyzed by a WST-1 assay. Vehicle-treated cells were set 100%. Mean ± SD. * *p* < 0.05 versus vehicle (*n* = 4). (**B**) Pericytes were treated as described in (**A**) and the cytotoxicity was assessed by a LDH assay. Vehicle-treated cells were used as control and set 100% (*n* = 4). Cells were permeabilized with Triton-X100 (0.1%) and used as high toxicity control (HC) for the LDH assay. Mean ± SD. * *p* < 0.05 versus vehicle (*n* = 4). (**C**) Pericytes were cultured for 24 h and thereafter treated with vehicle (DMSO) or CX-4945 (10 µM). The cell number was determined 0, 24 and 48 h after the onset of treatment. Vehicle-treated pericytes (0 h) were set 100% (*n* = 3). Mean ± SD. (**D**) Pericytes were treated as described in (**A**) and the MFI of BrdU-positive cells (% of total gated cells) was assessed by flow cytometry (*n* = 4). Mean ± SD. (**E**) Pericytes were pretreated for 48 h with vehicle (DMSO) or CX-4945 (10 µM) and analyzed by scratch assays (scale bar: 200 µm) and transwell migration assays (scale bar: 50 µm) at the indicated time points. For sprouting assays, spheroids were treated with vehicle (DMSO) or CX-4945 (10 µM) for 48 h and cumulative sprout length was analyzed (scale bar: 200 µm). (**F**) Quantitative analysis of gap area after 0, 16 and 24 h (scratch assay). The gap area at 0 h was set 100%. Mean ± SD. * *p* < 0.05 versus vehicle (*n* = 4). (**G**) Quantitative analysis of migrated cells after 5 h (transwell migration assay). Vehicle-treated cells were set as 100%. Mean ± SD. * *p* < 0.05 versus vehicle (*n* = 4). (H) Quantitative analysis of cumulative sprout length after 48 h (spheroid sprouting assay). The cumulative sprout length of vehicle-treated spheroids was set as 100%. Mean ± SD. * *p* < 0.05 versus vehicle (*n* = 4).

CX-4945 is the first CK2 inhibitor in clinical trials (ClinicalTrials.gov Identifier: NCT02128282). In addition to its pro-apoptotic activity, this drug has been shown to exert anti-angiogenic properties [10]. Therefore, we analyzed the effect of CX-4945 on the intrinsic migratory and networking ability of pericytes because these processes crucially support angiogenesis. To exclude that the observed effects are mediated by cytotoxicity of CX-4945, we performed different viability assays (Figure 3A–D). WST-1 assays showed a significantly reduced mitochondrial activity of CX-4945-treated pericytes when compared to vehicle-treated controls (Figure 3A). However, LDH assays revealed that this concentration is not cytotoxic, which could be confirmed by additional growth curves and BrdU assays (Figure 3C,D). The effect of CK2 inhibition on the proliferative and migratory activity of pericytes was determined by means of a scratch assay. Treatment of the cells with CX-4945 significantly reduced their proliferation and migration, as indicated by a larger gap area 16 and 24 h after scratching when compared to vehicle-treated controls (Figure 3E,F). To verify the inhibitory effect of CK2 inhibition on cell migration, we performed transwell assays. From this, we could show that CK2 inhibition significantly reduces the migratory capacity of pericytes (Figure 3E,G). Moreover, we generated spheroids consisting of human pericytes and assessed their cellular sprouting capacity. In line with the results of the scratch and transwell assays, CX-4945 markedly decreased the length of newly developing sprouts (Figure 3E,H).

### 3.4. Effect of CK2 Inhibition in Pericytes on Endothelial Tube Stabilization

**Figure 4:**
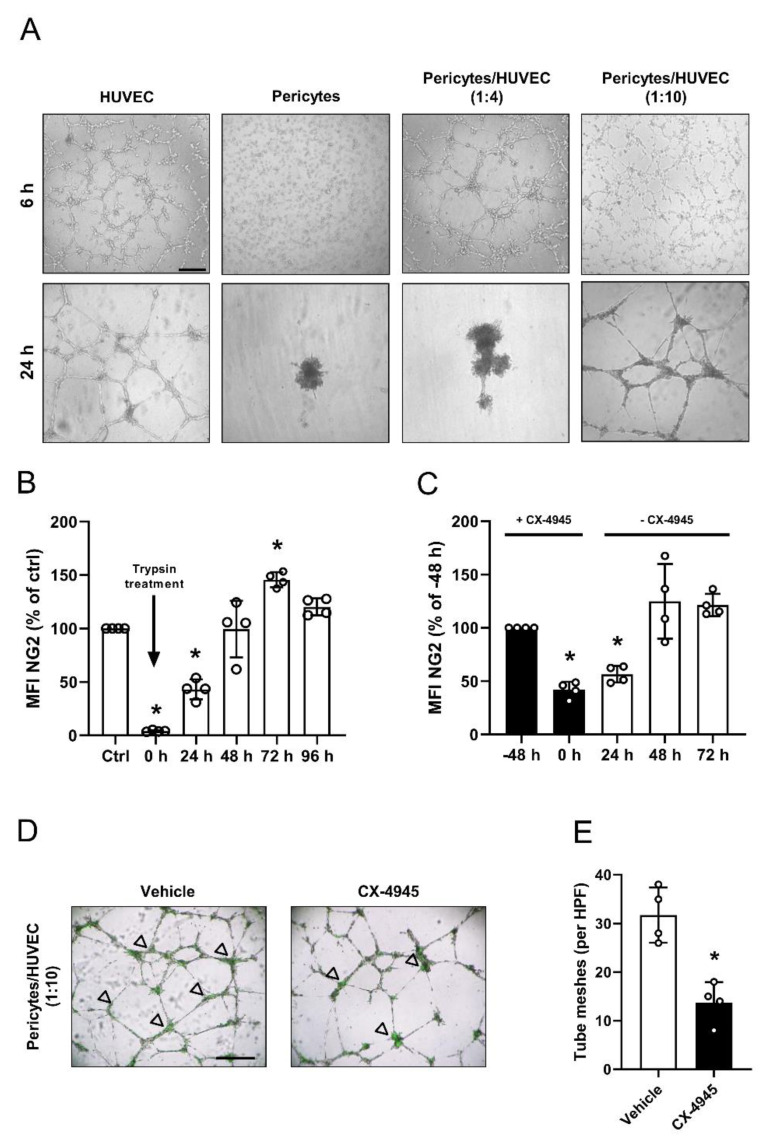
Effect of CK2 inhibition in pericytes on endothelial tube stabilization. (**A**) Tube formation assays were performed with HUVEC, pericytes as well as co-cultivated pericytes and HUVEC (ratio: 1:4 and 1:10). The formation of vessel-like structures was analyzed 6 and 24 h after seeding. Scale bar: 250 µm. (**B**) Pericytes were trypsinized and NG2 protein levels were detected by flow cytometry at the indicated time points. NG2 protein levels of scratched pericytes was set 100%. Mean ± SD. * *p* < 0.05 versus ctrl (*n* = 4). (**C**) Pericytes were treated with CX-4945 for 48 h (black bars) and subsequently incubated without CX-4945 (white bars) for the indicated time points. The NG2 protein levels were detected by flow cytometry. Pericytes at the time point -48 h were used as control and set 100%. Mean ± SD. * *p* < 0.05 versus -48 h (*n* = 4). (**D** and **E**) Pericytes were treated with vehicle (DMSO) or CX-4945 (10 µM) and cultivated for 48 h. Then, the pretreated pericytes were stained with a green fluorescence dye and co-cultivated with HUVEC (ratio: 1:10) (**D**). Arrows show green-stained pericytes at the branching points. Scale bar: 250 µm. Quantitative analysis of the number of tube meshes (per HPF) after 24 h (**E**). Mean ± SD. * *p* < 0.05 versus vehicle (*n* = 4).

In contrast to endothelial cells, pericytes do not form tubes in the tube formation assay (Figure 4A). However, they are capable of stabilizing endothelial tube meshes on their branching sites. For this purpose, pericytes were co-cultivated with HUVEC in a ratio of 1:4 or 1:10. Of interest, we found that pericytes solely integrate in endothelial tube meshes when co-seeded in a ratio of 1:10 (Figure 4A). To perform co-culture experiments with CX-4945-pretreated pericytes, we determined the biosynthesis time of NG2. For this, the extracellular domain of NG2 was completely cleaved by trypsin and the recovery of full-length NG2 was analyzed by flow cytometry over time. We found that full-length NG2 levels are restored from 0% of control to ~40%, ~100%, ~140% and ~120% of control after 24, 48, 72 and 96 h, indicating that physiological protein expression takes ~48 h (Figure 4B). We next pretreated pericytes with CX-4945 for 48 h and then cultivated the cells without CX-4945 to assess the long-lasting inhibitory effect of CX-4945 on NG2 expression. Pretreatment of pericytes with CX-4945 reduced the NG2 protein levels by up to ~40% (Figure 4C). 

The following analysis of recovery of the full-length NG2 protein after the removal of CX-4945 revealed an increase of only ~15%, ~85%, and ~80% of control after 24, 48 and 72 h, resulting in overall NG2 protein levels of ~55%, ~125% and ~120% of control. This clearly demonstrates a long-lasting inhibitory effect of CX-4945 on NG2 expression (Figure 4C). Based on these findings, we used CX-4945-pretreated pericytes with endothelial cells in a ratio of 1:10, to investigate the effect on endothelial tube stabilization. As expected, pretreatment of pericytes significantly reduced the number of tube meshes when compared to vehicle-pretreated controls (Figure 4D,E). 

### 3.5. Effect of CK2 Inhibition in Pericytes on the Angiogenesis

**Figure 5:**
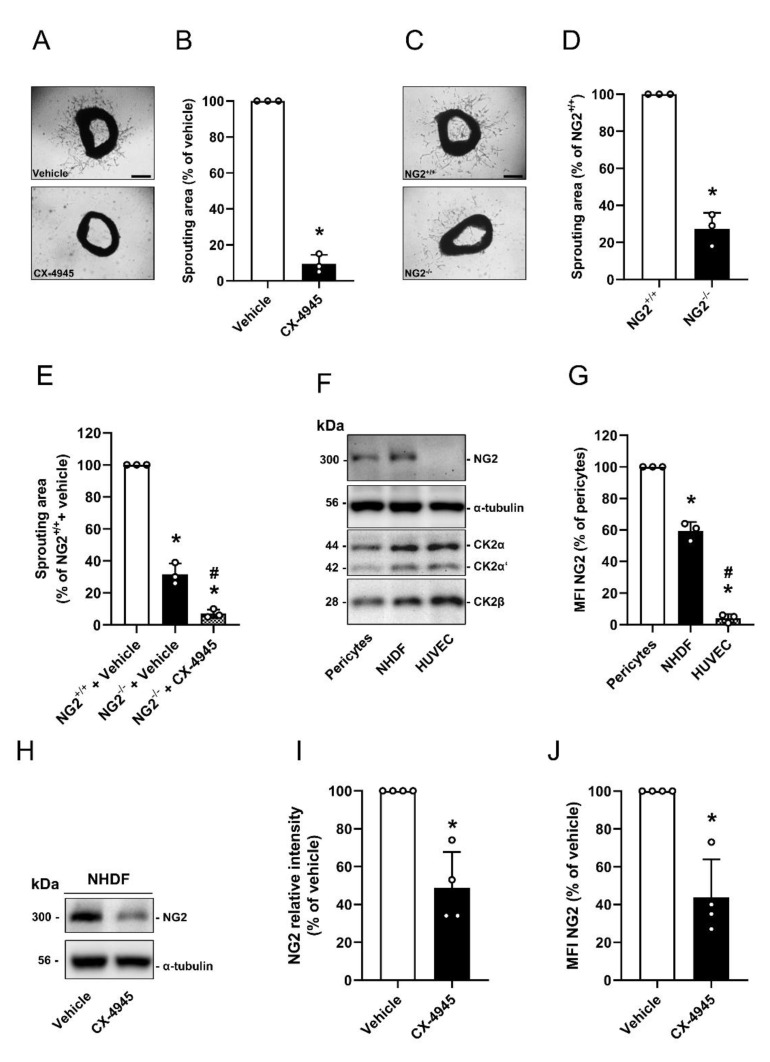
Effect of CK2 inhibition on the angiogenic activity of pericytes. (**A** and **B**) Aortic rings of wild type mice (NG2^+/+^) were embedded in Matrigel and treated with vehicle (DMSO) or CX-4945 (10 µM) for 6 d and sprouting of vessel-like structures from the aortic wall into the surrounding Matrigel was analyzed (**A**). Scale bar: 300 µm. Quantitative analysis of the sprouting area after 6 d (**B**). Vehicle-treated rings were set 100%. Mean ± SD. * *p* < 0.05 versus vehicle (*n* = 3). (**C** and **D**) Aortic rings of NG2^+/+^ and NG2^−/−^ mice were embedded in Matrigel for 6 d and the sprouting of vessel-like structures from the aortic wall into the surrounding Matrigel was analyzed (**C**). Scale bar: 300 µm. Quantitative analysis of sprouting area after 6 d (**D**). NG2^+/+^ aortic rings were set 100%. Mean ± SD. * *p* < 0.05 versus NG2^+/+^ (*n* = 3). (**E**) Aortic rings of NG2^+/+^ and NG2^−/−^ mice were embedded in Matrigel and treated as described in (**A**). Quantitative analysis of the sprouting area after 6 d. Vehicle-treated rings (NG2^+/+^) were set 100%. Mean ± SD. * *p* < 0.05 versus vehicle (NG2^+/+^), ^#^P < 0.05 versus vehicle (NG2^−/−^), (*n* = 3). (**F**) Pericytes, NHDF and HUVEC were lysed and the expression of NG2, CK2α, CK2α’, CK2β and α-tubulin (as loading control) was analyzed by Western blot. (**G**) Pericytes, NHDF and HUVEC were scratched and the MFI of NG2-positive cells was assessed by flow cytometry. Pericytes were set 100%. Mean ± SD. * *p* < 0.05 versus pericytes, ^#^ *p* < 0.05 versus NHDF (*n* = 3). (**H**–**J**) NHDF were treated with CX-4945 (10 µM) or vehicle (DMSO) for 48 h. The cells were lysed and the expression of NG2 and α-tubulin (as loading control) was analyzed by Western blot (**H**). NG2/α-tubulin was assessed by quantitative analysis of Western blots (**I**). The MFI of NG2-positive NHDF was detected by flow cytometry (**J**). Vehicle-treated cells were set as 100%. Mean ± SD. * *p* < 0.05 versus vehicle (*n* = 4).

Based on our in vitro results, we next investigated the effect of CK2 inhibition on the angiogenic activity of pericytes by means of ex vivo aortic ring assays. We treated aortic rings from NG2^+/+^ mice with CX-4945 or vehicle. Treatment with the CK2 inhibitor markedly suppressed the outgrowth of vascular sprouts from aortic rings when compared to vehicle-treated controls (Figure 5A,B). The comparison of aortic rings from NG2^+/+^ and NG2^−/−^ mice further showed that the loss of NG2 significantly reduced aortic sprouting (Figure 5C,D). Additional treatment of aortic rings from NG2^−/−^ mice with CX-4945 strengthened the anti-angiogenic effect of NG2 loss (Figure 5E). 

Vessel sprouts consist of endothelial cells, pericytes and fibroblasts [33]. It is known that the latter also support angiogenesis and are positive for NG2 [34,35,36]. Our Western blot and flow cytometric analyses revealed that NHDF express NG2 (Figure 5F,G) and inhibition of CK2 significantly decreases NG2 expression (Figure 5H–J). Hence, the anti-angiogenic effect of CK2 inhibition may not only be caused by the reduced NG2 expression on pericytes, but also by the reduction in NG2 expression on fibroblasts. 

**Figure 6:**
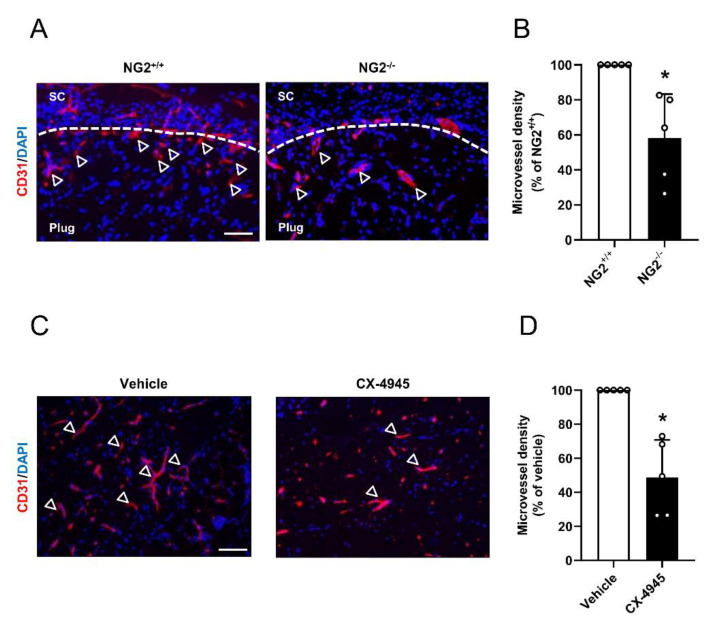
(**A**) Immunohistochemical detection of murine CD31-positive cells in empty Matrigel plugs of NG2^+/+^ and NG2^−/−^ mice after 7 d. Arrows show CD31-positive blood vessels; dotted lines represent edge of the plugs; SC = subcutaneous tissue. Scale bar: 150 µm. (**B**) Quantitative analysis of the microvessel density of Matrigel plugs after 7 d. Matrigel plugs of NG2^+/+^ mice were set as 100%. Mean ± SD. * *p* < 0.05 versus NG2^+/+^ (*n* = 5). (**C**) Immunohistochemical detection of human CD31 positive-cells in Matrigel plugs containing HUVEC mixed with vehicle- or CX-4945-treated pericytes in a ratio 10:1 after 7 d. Arrows show CD31-positive blood vessels. Scale bar: 150 µm. (**D**) Quantitative analysis of the microvessel density of Matrigel plugs after 7 d. Matrigel plugs containing vehicle-treated pericytes were set 100%. Mean ± SD. * *p* < 0.05 versus vehicle (*n* = 5).

Finally, we confirmed our results by an in vivo Matrigel plug assay. We first injected Matrigel plugs without cells into the flank of NG2^+/+^ and NG2^−/−^ mice to investigate the angiogenic potential of NG2 knockout mice. As expected, we determined a markedly decreased microvessel density in Matrigel plugs of NG2^−/−^ mice after 7 days when compared to controls (Figure 6A,B). Next, we assessed the effect of CK2 inhibition in pericytes on angiogenesis. For this purpose, pericytes were treated with vehicle or CX-4945 for 48 h and subsequently mixed in Matrigel with HUVEC in a ratio of 1:10. The cell suspension was injected subcutaneously into the flank of immunodeficient CD1 nu/nu mice. Immunohistochemical analyses of the Matrigel plugs 7 days after implantation revealed a significantly reduced density of CD31-positive microvessels in plugs with CX-4945-treated pericytes when compared to controls (Figure 6C,D). 

## 4. Discussion

In the present study, we analyzed, for the first time, the effect of CK2 inhibition on the angiogenic activity of human pericytes. We found that CK2 inhibition significantly diminishes the expression of the surface protein NG2. Gene regulatory analyses revealed that CK2 inhibition decreases the activity of a transcriptional active region close to the translational start of NG2. In addition, the suppression of CK2 activity in pericytes markedly reduces the formation of human microvessels in vitro and in vivo. Taken together, these findings indicate that CK2 is a crucial regulator of NG2-mediated angiogenic activity of pericytes.

Pericytes have a leading function in supporting endothelial sprouting and blood vessel formation. Signal transduction via angiopoietin-1 (Ang-1)/Tie-2 is one of the best characterized paracrine loops and has been shown to regulate endothelial maturation, stability and vascular leakage [13]. In recent decades, further surface proteins were identified on pericytes contributing to the angiogenic potential of these cells, including NG2 [13,16,17,18]. In this study, we detected a significantly reduced expression of NG2 in pericytes after CK2 inhibition. This was achieved both by pharmacological inhibition and knockdown of the catalytic CK2 subunits. Further analyses revealed that CK2 inhibition decreases NG2 gene expression. 

The regulatory mechanisms of NG2 expression are still largely unknown [37]. To date, only a few transcription factors have been identified, which activate or repress NG2 gene expression. Sellers et al. [32] characterized the mouse NG2 promoter by generating truncated promotor fragments. However, the human NG2 promoter region varies from that of mouse NG2 due to the different chromosomal localization. Accordingly, we herein characterized the human NG2 promoter for the first time. We determined a 2677 bp region upstream of the transcriptional start. The generation of overlapping truncated fragments of this region revealed a 114 bp transcriptional active region (P1.2.4.1). Of interest, CK2 inhibition significantly decreased the transcriptional activity of this region. However, CK2 cannot directly affect gene expression. Hence, we assume that the kinase may regulate NG2 expression via transcription factors. In silico analyses demonstrated that the sequence of P1.2.4.1 contains a bona-fide SP1 consensus sequence. This transcription factor can activate or repress transcription in response to physiological and pathological stimuli [38]. Moreover, SP1 is a substrate of CK2 and the loss of the phosphorylation increases its DNA-binding capacity [39,40]. Based on these findings, it is tempting to speculate that CK2 inhibition increases the ability of SP1 to repress NG2 transcription. 

Promoters could cover a large region upstream of genes, which harbors enhancer as well as silencer regions [41]. Therefore, it should be noted that silencer regions might be crucially involved in NG2 gene expression. In fact, we detected decreased activity of NG2^P2^ when compared to pGL4-basic, indicating that silencer elements upstream of NG2^P1^ may influence NG2 expression. However, whether these elements are involved in NG2 expression has yet to be clarified by further studies. 

Cell proliferation, migration and sprouting capacity are important steps in the development of new microvessels. It is well known that these steps are affected in endothelial cells after CK2 inhibition [10]. The effects of CK2 inhibition in pericytes have not been studied to date, although these perivascular cells are known to be involved in angiogenesis [12]. Upon angiogenic stimulation, pericytes migrate from the wall of mature blood vessels, and thus allow endothelial cells to form angiogenic sprouts [14]. Ozerdem et al. [25] showed that knockout of NG2 results in a decreased neovascularization of retinal tissue after ischemia. Hence, we speculated that the reduced NG2 expression after CK2 inhibition may decrease the angiogenic potential of pericytes. In fact, the treatment of pericytes with CX-4945 significantly inhibited their proliferation, migration and sprouting capacity. 

Mono-cultured pericytes are not capable of forming vessel-like structures in contrast to endothelial cells [42]. However, pericytes have a stabilizing effect on endothelial tubes [43]. To test this, we co-cultured pericytes with HUVEC in the ratio of 1:4 and 1:10. Our results revealed that pericytes are integrated into tube meshes when using a ratio of 1:10, whereas a ratio of 1:4 disturbed tube formation. This could be explained by the fact that in vitro mono-cultured pericytes form spheroids, and thus the high cell number in a ratio of 1:4 could promote, under these in vitro conditions, the formation of spheroidal structures instead of tube stabilization [42]. However, it should be noted that the tube formation assay does not reflect the physiological situation in vivo, in which blood vessels are embedded in the stabilizing ECM. In addition, the pericytes are not capable of forming spheroidal structures in vivo, hence ratios of pericytes to endothelial cells from 1:1 to 1:100 can be found depending on the tissue [44].

We further assessed the effect of CK2 inhibition on blood vessel development by means of aortic ring assays. Treatment of aortic rings from wild type mice with CX-4945 efficiently prevented sprout formation. This is not surprising considering the fact that the CK2 inhibitor has previously been shown to suppress the proliferation, migration and tube formation of endothelial cells [10]. Next, we compared the angiogenic sprouting activity of aortic rings from NG2^+/+^ and NG2^−/−^ mice and analyzed the sprouting activity of aortic rings from NG2^−/−^ mice in the presence of CX-4945. We could demonstrate that NG2 knockout also decreases vessel sprouting. Previous studies have shown that NG2 loss in genetically modified mice does not only directly suppress angiogenesis but also the number of pericytes covering newly formed microvessels [45,46]. Although not analyzed in the present study, it may be interesting to clarify whether the latter mechanism contributed to the herein observed reduced sprouting activity of aortic rings from NG2^−/−^ mice. Our aortic ring assays additionally demonstrated that the reduced vessel sprouting in NG2^−/−^ mice is further strengthened by the additional inhibition of CK2. This result indicates that CK2 inhibition has additive NG2-independent effects on angiogenesis. 

Beside endothelial cells and pericytes, fibroblasts also contribute to angiogenesis. These cells support the angiogenic process by synthesis and maintenance of the ECM [34] and by releasing pro-angiogenic factors [35]. We herein found that CX-4945 decreases the NG2 expression not only in pericytes but also in fibroblasts. Hence, the reduced NG2 protein levels in fibroblasts may contribute to reduced angiogenesis after CK2 inhibition.

In vivo, we found a significantly reduced microvessel ingrowth into empty Matrigel plugs of NG2^−/−^ mice when compared to NG2^+/+^ mice. This is in line with previous studies showing a decreased pericyte proliferation and motility in NG2^−/−^ mice leading to diminished numbers of blood vessels [25,45]. To study the effect of downregulated NG2 expression after CK2 inhibition, we prepared Matrigel plugs containing CX-4945-treated pericytes in combination with untreated HUVEC. Of interest, we detected a significantly lower microvessel density after CK2 inhibition. This may be caused by decreased NG2 expression during the initial angiogenic phase, as it takes ~48 h to restore NG2 protein levels after treatment with CX-4945. These findings support the view that CK2 regulates the NG2-mediated angiogenic activity of pericytes.

CK2 phosphorylates more than five hundred proteins [2]. Hence, beside NG2, further proteins in pericytes are regulated by CK2. For instance, Di Maira et al. [47] have demonstrated that the inhibition of CK2 reduces Akt phosphorylation on serine 129. This, in turn, reduces the activity of transcription factor forkhead box (FOXO)3A [48]. Of interest, Teichert et al. [49] recently identified FOXO3A as an important downstream molecule of Tie2-mediated Akt signal transduction in pericytes. They found that autocrine stimulation of pericytes with Ang1 attenuates FOXO3A activity, which promotes the maturation of blood vessels [49]. Because vessel maturation counteracts angiogenesis [50], it is tempting to speculate that CK2 inhibition also suppresses blood vessel development by lowering FOXO3A activity in pericytes. 

Besides the well-described angiogenic function of CK2 in endothelial cells [10], we herein demonstrate that CK2 inhibition reduces the proliferation and migration of pericytes, resulting in a decreased angiogenesis. Further insights into the regulatory mechanism revealed that this is due to a diminished NG2 gene expression. Of interest, NG2 is not only expressed in pericytes but also in other cells, such as oligodendrocyte progenitor cells [51] and several different cancer cells [52]. However, how CK2 inhibition affects NG2-mediated functions in these cells needs to be clarified by further studies. 

## Supplementary Materials

The following are available online at https://www.mdpi.com/2073-4409/9/6/1546/s1, Table S1: List of primers
