# Supplementary material for: Protein Kinase CK2 Regulates Nerve/Glial Antigen (NG)2-Mediated Angiogenic Activity of Human Pericytes
Source: Cells. 2020 Jun 25;9(6):1546. doi: 10.3390/cells9061546 (PMC7348826; doi:10.3390/cells9061546)
Supplement: Supplementary file 1 [file cells-09-01546-s001.pdf]

Table 1. List of primers.

Primer qRT-PCR	Sequenz 5′ – 3′	Amplicon length [bp]
NG2-fw<br>NG2-rv	GCAAGCCGATGTGGATTC<br>ATGGCGGATGGTAGGATG	141
GAPDH-fw<br>GAPDH-rv	CCACCCATGGCAAATTCC<br>ACTCCACGACGTACTCAG	141
β-Aktin-fw<br>β-Aktin-rv	TCCCTGGAGAAGAGCTACGA<br>AGCACTGTGTTGGCGTACAG	194
**Primer NG2 promoter**	**Restriction site Sequenz 5′ – 3′**	**Amplicon length [bp]**
P2-fw<br>P2-rv	AGACTC GAGCTCTGGTTTCAAGAC<br>AGAAAG CTTCACCTCAGTCTCCCT	1382
P1-fw<br>P1-rv	AGACTC GAGCTCTGGTTTCAAGAC<br>AGAAAG CTTAGGACTTGCGAGGAG	1369
P1.1-fw<br>P1.1-rv	AGACTC GAGTTGAGCTGCACTTTC<br>AGAAAG CTTAGGACTTGCGAGGAG	765
P1.2-fw<br>P1.2-rv	AGACTC GAGCTCAGTTTCCTCATC<br>AGAAAG CTTCCCTAAGTGCAGTGC	762
P1.2.1-fw<br>P1.2.1-rv	AGACTC GAGTTGAGCTGCACTTTC<br>AGAAAG CTTACTCCAGAGGCACCA	236
P1.2.2-fw<br>P1.2.2-rv	AGACTC GAGGACTGAGGTGAGGCCCTTC<br>AGAAAG CTTCCCTACTTCCCTCCTTTCTG	237
P1.2.3-fw<br>P1.2.3-rv	AGACTC GAGTCTCCTGAACTCCCGCAAAG<br>AGAAAG CTTCTCGGGTTTCAGGCTTGGAC	276
P1.2.4-fw<br>P1.2.4-rv	AGACTC GAGCGGGCCCTTTAAGAAGGTTG<br>AGAAAG CTTAGGACTTGCGAGGAG	201
P1.2.4.1-fw<br>P1.2.4.1-rv	AGACTC GAGCGGGCCCTTTAAGAAGGTTG<br>AGAAAG CTTGGTGTCCGCGCACTTAACTC	114
P1.2.4.2-fw<br>P1.2.4.2-rv	AGACTC<br>GAGGAGTTAAGTGCGCGGACACC<br>AGAAAG CTTAGGACTTGCGAGGAG	107
pGL4.10 [luc2]-fw<br>pGL4 [luc2]-rv	CCAGTG CAAGTGCAGGTGCCAGAAC<br>GAATGG CGCTGGGCCCTTCTTAATG	
**Primer genotype**	**Sequenz 5′ – 3′**	**Amplicon length [bp]**
NG2wt-fw<br>NG2wt-rv	GGCAAACCCAGAGCCCTGCC<br>GCTGGAGCTGACAGCGGGTG	557
NG2wt-fw<br>NG2CreERT-rv	GGCAAACCCAGAGCCCTGCC<br>GCCCGGACCGACGATGAAGC	829
